# Distally Based Adipofascial Flaps for Reconstruction of Distal Lower-Extremity Defects: Clinical Outcomes and Reconstructive Considerations

**DOI:** 10.3390/jcm15197402

**Published:** 2026-09-23

**Authors:** Ugur Horoz, Hülda Rifat Ozakpinar, Emre Inozu, Ergin Seven, Avni Tolga Eryilmaz, Ali Teoman Tellioglu

**Affiliations:** 1 Private, Plastic Reconstructive and Aesthetic Surgery Clinic, 34365 Istanbul, Türkiye; 2 Plastic Reconstructive and Aesthetic Surgery Department, Lokman Hekim University, 06510 Ankara, Türkiye; 3 Private, Plastic Reconstructive and Aesthetic Surgery Clinic, 06510 Ankara, Türkiye; dremre78@gmail.com (E.I.); mdtolgaer@yahoo.com (A.T.E.); aliteoman.tellioglu@gmail.com (A.T.T.)

**Keywords:** adipofascial flap, distal lower extremity, reconstructive surgical procedures, soft tissue defects, sural flap

## Abstract

**Background:** Soft-tissue defects of the distal lower extremity involving the malleolar regions, the Achilles tendon area, and the heel remain challenging to reconstruct because of limited local tissue availability and variable perforator-based vascularity. Distally based adipofascial flaps, which omit the cutaneous paddle used in conventional fasciocutaneous flap designs, may provide a useful option for coverage of selected defects in this region while avoiding the need for microsurgical free-tissue transfer. **Methods:** We retrospectively reviewed 24 patients who underwent reconstruction of distal lower-extremity defects with distally based adipofascial flaps. Demographic, clinical, operative, and postoperative outcome data were collected, including flap survival, wound healing, and postoperative complications. Flap survival was assessed with separate consideration of total flap loss and partial distal flap-edge necrosis. **Results:** Defects involved the heel (37.5%), lateral malleolus (20.8%), medial malleolus (20.8%), and Achilles tendon region (20.8%). Six patients (25.0%) had diabetes mellitus, and four (16.7%) had an underlying vascular disorder. No total flap loss occurred. Limited distal flap-edge necrosis occurred in five patients (20.8%), while wound dehiscence occurred in three (12.5%) and local infection in two (8.3%). The observed complications were managed with conservative treatment or minor secondary procedures, including debridement and skin grafting when required. **Conclusions:** In this retrospective case series, distally based adipofascial flaps provided soft-tissue coverage for selected small-to-medium defects of the distal lower extremity, with no total flap loss despite a 20.8% rate of limited distal flap-edge necrosis. These findings suggest that this technique may be a useful reconstructive option in appropriately selected patients, although its comparative effectiveness and safety relative to other reconstructive methods require further evaluation in comparative studies.

## 1. Introduction

The distal third of the lower extremity is one of the most challenging anatomical regions in reconstructive surgery because of its limited soft-tissue reserve, thin and relatively immobile skin envelope, and precarious vascularity coursing close to bone, tendon, and neurovascular structures [1]. Defects in this region arise from a variety of etiologies, including high-energy trauma, open fractures, chronic osteomyelitis, tumor resection, and diabetic foot wounds, and frequently cannot be closed with simple skin grafting because of exposed bone or tendon, necessitating vascularized tissue transfer [1].

Free tissue transfer is widely regarded as the gold standard for reconstructing large, complex distal leg defects [2,3]; however, it carries important limitations, including the need for microsurgical expertise and equipment, prolonged operative and recovery times, donor-site morbidity, and potential risks in patients with comorbidities such as peripheral arterial disease, diabetes, or advanced age [2,3]. Local flap options that avoid microvascular anastomosis and are technically less demanding therefore remain valuable alternatives in appropriately selected patients.

Distally based fasciocutaneous flaps occupy an important place in this context. Donski and Fogdestam first described a distally based fasciocutaneous flap from the sural region in 1983 [4], and Masquelet et al. subsequently detailed the neurocutaneous island flap based on the vascular axis of the sural nerve in 1992, contributing to its widespread clinical adoption [5]. The distally based sural flap has since become an established option for reconstructing the distal leg, ankle, and heel because of its retrograde perfusion and technical simplicity; however, complications such as venous congestion and partial or complete flap necrosis have been reported at variable rates, prompting numerous modifications intended to optimize flap performance [6,7].

One such modification is the adipofascial variant, which, unlike the classic fasciocutaneous design, is elevated without a cutaneous paddle, transferring only the subcutaneous fat and deep fascia. Lin et al. described a series of medial, lateral, and saphenous artery-based adipofascial flap variants during the 1990s [8,9,10], and Lee et al. subsequently reported a lateral adipofascial flap based on the peroneal artery perforator [11]. Omitting the skin island may allow a more pliable flap and pedicle configuration, facilitate coverage of anatomically constrained defects, and, in selected cases, permit primary closure of the donor site. When required, a skin graft can be applied over the adipofascial flap for definitive wound coverage. In a comparative series of 145 patients, Schmidt et al. reported a shorter operative time and more favorable aesthetic outcomes with the adipofascial variant compared with the classic fasciocutaneous sural flap [12], while a subsequent 104-patient follow-up series from the same group reported favorable clinical outcomes with the technique for distal leg reconstruction [13].

The contemporary literature reflects an expanding range of applications for distally based adipofascial flaps, with favorable outcomes reported for oncologic foot and ankle defects [14], pediatric patients [15], combined use with acellular dermal matrix [16], and modifications based on a single sural nerve branch to limit sensory loss [17]. Comparative studies involving alternative local options, including bipedicled flaps [18] and the gastrocnemius musculoadipofascial flap [19], further underscore the need for a multifaceted approach to flap selection in distal leg reconstruction [20].

Collectively, the available evidence suggests that distally based adipofascial flaps may provide a useful reconstructive option for selected small-to-medium soft-tissue defects of the distal lower extremity, particularly in patients for whom free tissue transfer is not required or is not feasible. However, outcomes may vary according to defect characteristics, flap design, vascular basis, patient factors, and postoperative management, and reported complication rates are not directly comparable across studies because of differences in study populations and outcome definitions. Further clinical studies with systematic reporting of flap survival, partial necrosis, wound healing, and other postoperative outcomes are warranted. The present study aimed to evaluate the clinical outcomes and reconstructive considerations of distally based adipofascial flaps used for reconstruction of distal lower-extremity defects.

## 2. Materials and Methods

### 2.1. Study Design and Setting

This retrospective case series evaluated the clinical outcomes of distally based adipofascial flaps used to reconstruct soft-tissue defects of the distal lower extremity at Ankara Dışkapı Yıldırım Beyazıt Training and Research Hospital between January 2012 and May 2017. The study was conducted in accordance with the principles of the Declaration of Helsinki and reported according to the STROBE (Strengthening the Reporting of Observational Studies in Epidemiology) guidelines for observational studies.

### 2.2. Patient Selection

Patients were eligible for inclusion if they were 18 years of age or older and had undergone reconstruction of a distal lower-extremity soft-tissue defect using a distally based adipofascial flap, with adequate operative and postoperative follow-up records available for review. Patients were excluded if the defect was located outside the distal lower extremity, if reconstruction had been performed using an alternative flap technique, or if the available clinical documentation was insufficient for outcome assessment. The patient selection process is summarized in the study flow diagram (Figure 1).

Indications for reconstruction included acute trauma, chronic non-healing wounds, infection, and defects following tumor excision, particularly when tendon, bone, or neurovascular structures were exposed. All defects involved the medial malleolus, lateral malleolus, Achilles tendon region, or heel.

### 2.3. Data Collection

Demographic and clinical data were retrospectively extracted from institutional records, including age, sex, smoking status, presence of diabetes mellitus, and underlying vascular disease. Defect-related variables included etiology, anatomical location, dimensions, and the specific anatomical structures exposed at presentation. Operative variables included flap dimensions, duration of hospitalization, and duration of postoperative immobilization.

### 2.4. Surgical Technique

The same fundamental surgical technique was applied consistently in all 24 patients. All procedures were performed under general anesthesia with tourniquet control. Distal fasciocutaneous perforators were identified preoperatively using a handheld acoustic Doppler device. The flap was designed to preserve the identified distal perforators and maintain adequate vascular inflow to the adipofascial tissue.

A lazy-S or zigzag incision was marked along the central posterior leg, extending from the proximal margin of the planned flap toward the defect. Following skin incision, dissection proceeded from proximal to distal through the subcutaneous tissue overlying the gastrocnemius fascia, with care taken to preserve the subdermal vascular plexus and maintain adequate skin-flap thickness.

The adipofascial flap was elevated immediately superficial to the muscular fascia without formal isolation or skeletonization of a named vascular pedicle. The flap was therefore maintained on its distal perforator-based vascular supply rather than on an individually dissected named arterial pedicle. Dissection was stopped 3–4 cm proximal to the upper margin of the defect to protect the distal fasciocutaneous perforators supplying the flap and to establish the pivot point for transposition. Flap elevation was performed primarily using tissue scissors, while bipolar electrocautery was used for hemostasis to minimize the risk of thermal injury associated with monopolar devices. Following tourniquet release, flap perfusion was assessed and meticulous hemostasis was achieved. Proximal perforators encountered and divided during flap elevation were ligated or coagulated, whereas the previously identified distal perforators were preserved.

The flap was then transposed into the defect without tension, torsion, or compression at the pivot point and secured to the recipient-site margins with absorbable sutures. A small-caliber suction drain was placed beneath the flap. The donor site was closed primarily, and the exposed adipofascial surface was covered with a split-thickness skin graft harvested from the ipsilateral thigh and secured to achieve close contact with the vascularized adipofascial bed.

Representative reconstruction is illustrated in Figure 2.

### 2.5. Outcome Measures

The primary outcome was total flap loss, defined as complete necrosis of the transferred adipofascial tissue requiring flap removal. Partial distal flap-edge necrosis was assessed separately as a secondary outcome and was not considered total flap loss.

Secondary outcomes included skin-graft take, wound healing, venous congestion, postoperative infection, wound dehiscence, distal flap-edge necrosis, donor-site morbidity, need for secondary surgical intervention, and long-term reconstructive outcome.

### 2.6. Statistical Analysis

Continuous variables are presented as means with ranges, and categorical variables as frequencies with percentages. Given the descriptive nature of this retrospective case series and the absence of a comparator group, no formal comparative statistical testing was performed. Statistical analyses were conducted using SPSS version 25.0 (IBM Corp., Armonk, NY, USA).

### 2.7. Ethical Considerations

This study was conducted in accordance with the principles of the Declaration of Helsinki and was approved by the Ethics Committee of Ankara Bilkent City Hospital (approval number: E2/26/16243; date: 19 August 2026). Given the retrospective design and use of anonymized clinical data, the requirement for individual informed consent for study participation was waived by the ethics committee. Separate written consent was obtained from patients for the publication of identifiable clinical photographs.

## 3. Results

The study included 24 patients who underwent reconstruction of distal lower-extremity defects using distally based adipofascial flaps. Demographic and clinical characteristics are summarized in Table 1. The study comprised 21 men (87.5%) and 3 women (12.5%), with ages ranging from 30 to 64 years (mean, 39 years). Six patients (25.0%) had diabetes mellitus, and four (16.7%) had an underlying vascular disorder. Defect etiology was chronic wound in 11 patients (45.8%), trauma in 8 (33.3%), infection in 3 (12.5%), and tumor excision in 2 (8.3%). Defect dimensions ranged from 2.1 to 5.8 cm, duration of hospitalization ranged from 2 to 7 days, and postoperative immobilization ranged from 14 to 21 days. Representative clinical cases are shown in Figure 3, Figure 4, Figure 5 and Figure 6.

The anatomical distribution of the defects is presented in Table 2. The heel was the most frequently affected site (*n* = 9, 37.5%), followed by the lateral malleolus, medial malleolus, and Achilles tendon region, with 5 cases each (20.8%).

Postoperative outcomes and complications are summarized in Table 3. The overall follow-up period was at least 12 months for all patients. During follow-up, no total flap loss was observed. Limited distal flap-edge necrosis occurred in 5 patients (20.8%); 3 patients required debridement followed by split-thickness skin grafting, whereas 2 patients healed by secondary intention. Wound dehiscence requiring secondary suturing occurred in 3 patients (12.5%), and postoperative local infection occurred in 2 patients (8.3%). The infections resolved with local wound care and oral antibiotics without further surgical intervention. The representative cases demonstrated durable soft-tissue coverage and satisfactory wound healing at 10–18 months of follow-up.

## 4. Discussion

The distal third of the lower extremity, including the malleolar regions, Achilles tendon area, and heel, remains a challenging reconstructive area because of its limited soft-tissue availability, relatively immobile skin, and proximity to critical osseous, tendinous, and neurovascular structures [1]. In the present retrospective case series, no total flap loss occurred among the 24 reconstructions. However, limited distal flap-edge necrosis developed in 5 patients (20.8%), indicating that the absence of total flap loss should be distinguished from partial tissue loss when evaluating the reliability of this technique. Three patients with distal flap-edge necrosis required debridement followed by split-thickness skin grafting, whereas two healed by secondary intention. Thus, the present findings suggest that distally based adipofascial flaps can provide effective coverage in selected distal lower-extremity defects, while partial distal necrosis remains a clinically relevant complication.

The rationale for using an adipofascial rather than a fasciocutaneous design in this anatomical region is primarily related to its tissue composition and vascular characteristics. Mojallal et al. proposed that venous drainage of the adipofascial flap may be more efficient than that of the fasciocutaneous variant because the draining venous network is predominantly located within the fascial plane [21]. This anatomical characteristic may reduce venous congestion, which has historically been an important contributor to complications in distally based flap reconstruction. In the present series, no clinically documented episode of venous congestion or total flap necrosis was observed. Nevertheless, the occurrence of distal flap-edge necrosis in 20.8% of patients indicates that the adipofascial design does not completely eliminate the risk of distal perfusion-related tissue loss.

The complication rate observed in the present series should be interpreted cautiously in comparison with previously published studies. Follmar et al., in a pooled analysis of classic fasciocutaneous distally based sural flaps, reported a combined partial or total necrosis rate of 14.3% [22], whereas higher overall complication rates have also been reported for island fasciocutaneous flaps [21]. Although the present series demonstrated no total flap loss, the rate of limited distal flap-edge necrosis was 20.8%, which is numerically higher than the combined necrosis rate reported by Follmar et al. [22]. Differences in flap design, patient selection, defect characteristics, vascular anatomy, outcome definitions, and follow-up periods make direct comparison difficult. Therefore, our findings do not establish that omission of the skin paddle improves the safety or reliability of the flap. Rather, they indicate that the adipofascial design may provide successful coverage without total flap loss in appropriately selected patients, although partial distal tissue loss remains possible.

Our findings can also be considered in the context of larger series evaluating risk factors for partial necrosis of distally based flaps. In an analysis of 435 distally based sural flaps, Peng et al. reported an overall partial-necrosis rate of 8.97%, which decreased from 13.41% in an earlier treatment era to 5.86% following subsequent technical refinements, underscoring the potential importance of surgical technique in flap reliability [23]. In a separate comparison of elderly patients (≥60 years) and younger patients, the same group reported partial-necrosis rates of 9.4% and 9.1%, respectively, with no significant difference between age groups [24]. These findings suggest that advanced age alone may not necessarily preclude successful distally based flap reconstruction when flap design and vascularity are carefully considered. In the present study, 6 patients (25.0%) had diabetes mellitus and 4 (16.7%) had an underlying vascular disorder, yet no total flap loss occurred. Nevertheless, the presence of distal flap-edge necrosis in this population emphasizes the need for careful patient selection and preoperative assessment of tissue perfusion. Demirtas et al. similarly emphasized the potential for increased complications in older and diabetic patients undergoing adipofascial flap reconstruction and recommended appropriate preoperative vascular assessment in this subgroup [25]. Dai et al. also reported that distally based flaps may remain useful for diabetic foot reconstruction when perfusion is carefully evaluated before surgery [26].

A further technical modification described in the literature involves incorporation of a thin cuff of gastrocnemius muscle at the base of the adipofascial pedicle to potentially increase vascular robustness. In a retrospective case–control study, Schmidt et al. reported a trend toward improved flap reliability when a muscle cuff was incorporated compared with the standard adipofascial pedicle, particularly in technically demanding reconstructions [27]. Although a muscle cuff was not routinely incorporated in the present series, this modification may be considered in selected cases in which perfusion is considered borderline or a longer pedicle is required. However, prospective comparative studies would be necessary to determine whether this modification provides a clinically meaningful advantage.

The anatomical distribution of defects in our series included the heel in 37.5% of cases, with the lateral malleolus, medial malleolus, and Achilles tendon region each accounting for 20.8%. These locations represent common reconstructive problems in the distal lower extremity because exposed bone, tendon, or orthopedic hardware may preclude simple skin grafting. Distally based adipofascial techniques based on different regional perforator systems have been described for coverage of these areas, providing relatively thin and pliable tissue that can conform to the contours of the foot and ankle [4,9,11]. Yeo et al. reported successful reconstruction of lateral malleolar defects using a peroneal artery perforator-based adipofascial turnover flap in a comorbid patient population, suggesting that this approach may be useful for selected lateral malleolar defects [28]. The lateral malleolus accounted for 20.8% of the defects in our series. Nevertheless, the relatively small defect dimensions in the present study (2.1–5.8 cm) should be considered when interpreting these findings. Adipofascial flaps are particularly applicable to small-to-medium soft-tissue defects, whereas larger defects involving extensive composite tissue loss may still require more extensive local or free-tissue reconstruction [2,3].

The broader adipofascial flap literature provides additional support for the use of this tissue concept in appropriately selected reconstructive situations. In a systematic review of 32 studies including 951 adipofascial flaps across different anatomical regions, D’Elia et al. reported an overall flap survival rate exceeding 95% and described favorable contour characteristics and low donor-site morbidity in lower-limb applications [29]. However, these findings represent heterogeneous anatomical sites, flap designs, and indications and therefore should not be directly extrapolated to the present cohort. They nevertheless provide biological and clinical support for the use of adipofascial tissue when a thin, pliable coverage option is desirable.

The recent series by Costa et al. provides a relevant contemporary clinical comparison for the present cohort [30]. In their retrospective study of 32 patients who underwent reconstruction of chronic defects of the lower third of the leg with the reverse sural neuroadipofascial flap, chronic skin ulcers were the most frequent etiology (40.6%), and the heel (34.4%) and the malleolar regions (21.9% combined) accounted for a substantial proportion of the defects. These indications closely resemble those of the present series, in which chronic wounds accounted for 45.8% of the defects and the heel was the most frequently involved site (37.5%), although their patients were considerably older (mean age, 62.2 vs. 39 years). Costa et al. reported early or late postoperative complications in six patients (18.8%), evaluated scar stability as an indicator of long-term wound durability, and concluded that the flap is a versatile and technically straightforward option for chronic defects of the distal leg, including both malleoli and the heel, owing to its long vascular pedicle [30]. In the present series, distal flap-edge necrosis alone occurred in 20.8% of patients; however, differences in flap design, patient age, defect etiology, and complication definitions preclude direct comparison of complication rates between the two series.

The biological rationale emphasized by Costa et al. is particularly relevant to the adipofascial concept. The authors highlighted the regenerative potential of the transferred vascularized adipose tissue, which is rich in stem cells, in the healing of complex wounds of the lower third of the leg [30]. Because the adipofascial flap transfers only vascularized subcutaneous fat and deep fascia, this mechanism may partly explain the durable soft-tissue coverage observed in our representative cases at 10–18 months of follow-up. Nevertheless, the regenerative contribution of the transferred adipose tissue was not directly assessed in the present study and remains to be confirmed. In addition, Sinha et al. prospectively evaluated the distally based sural artery adipofascial flap in 21 patients with distal leg and ankle defects, providing prospective data on adipofascial reconstruction in this anatomical region [31].

The comparison of adipofascial and muscle-based reconstruction reported by Lee and Chang is also relevant to the current findings. In their comparative study of 32 patients with distal lower-extremity defects, patients undergoing adipofascial reconstruction were older and had more comorbidities than those undergoing local muscle flap reconstruction, while flap survival, infection rates, and donor-site complications were comparable between groups; the adipofascial group also had a lower daily drainage volume [32]. These findings support consideration of adipofascial flaps as one of several reconstructive options in patients for whom a thin soft-tissue cover is desirable. However, because the present study did not include a comparator group, it cannot determine whether adipofascial flaps are superior to muscle flaps or free flaps with respect to survival, complications, functional outcomes, or donor-site morbidity.

Preoperative perforator mapping with a handheld acoustic Doppler device was performed in all cases in the present series. Such mapping may assist in identifying suitable distal perforators and planning flap elevation while limiting unnecessary dissection [33]. In combination with atraumatic tissue handling and avoidance of excessive tension, torsion, or compression during flap transposition, this approach may have contributed to the absence of total flap loss observed in our cohort. However, because this was a retrospective uncontrolled series, the independent contribution of Doppler mapping to the observed outcomes cannot be determined.

Postoperative wound dehiscence occurred in 3 patients (12.5%) and local infection in 2 patients (8.3%). These complications were managed with secondary suturing or local wound care and oral antibiotics, respectively, without the need for additional flap reconstruction. The absence of total flap loss despite these postoperative complications suggests that the observed adverse events were generally manageable within the reconstructive pathway. Nevertheless, the 20.8% incidence of distal flap-edge necrosis remains important because three patients required additional debridement and skin-graft coverage.

The relatively short hospitalization period (2–7 days) and postoperative immobilization period (14–21 days) observed in this series indicate that the procedure can be incorporated into a relatively limited postoperative treatment course in appropriately selected patients. However, these findings should not be interpreted as evidence that the technique is superior to free-tissue transfer in terms of hospitalization, recovery, cost, or resource utilization, as no comparator group was included in the present study. The practical advantages of the technique may be particularly relevant in situations in which a local or regional solution is appropriate and microsurgical reconstruction is unnecessary or unsuitable.

This study has several limitations. First, its retrospective, single-center design and modest sample size (*n* = 24) limit the generalizability of the findings and preclude robust analysis of risk factors for distal flap-edge necrosis. Second, the absence of a direct comparator group, such as a fasciocutaneous distally based flap, local muscle flap, or free flap, prevents definitive conclusions regarding comparative safety, reliability, aesthetic outcomes, or functional outcomes. Third, the observed 20.8% rate of distal flap-edge necrosis demonstrates that the absence of total flap loss should not be interpreted as complete freedom from flap-related complications. Fourth, long-term functional outcomes and validated patient-reported outcome measures were not systematically collected, limiting assessment beyond wound healing and reconstructive durability. Finally, differences in vascular anatomy, defect characteristics, and flap dimensions may influence outcomes and should be considered when extrapolating these findings to other patient populations.

In conclusion, this retrospective case series demonstrates that distally based adipofascial flaps can provide soft-tissue coverage for selected small-to-medium defects of the distal lower extremity, with no total flap loss observed among 24 patients. However, limited distal flap-edge necrosis occurred in 20.8% of cases, including three patients who required secondary debridement and skin-graft coverage. Accordingly, the present findings support the technique as a potentially useful reconstructive option rather than establishing superiority over other flap techniques. Careful patient selection, preoperative assessment of vascularity, appropriate flap design, and meticulous postoperative management remain important considerations. Comparative prospective studies with standardized functional and patient-reported outcomes are warranted to better define the role of distally based adipofascial flaps in distal lower-extremity reconstruction.

## 5. Conclusions

Distally based adipofascial flaps provided soft-tissue coverage for selected distal lower-extremity defects in this retrospective case series, with no total flap loss observed among the 24 patients. However, limited distal flap-edge necrosis occurred in 20.8% of cases, including three patients who required debridement followed by split-thickness skin grafting. These findings suggest that distally based adipofascial flaps may represent a useful reconstructive option for selected defects involving the malleolar, Achilles tendon, and heel regions, particularly when thin and pliable soft-tissue coverage is desired. However, the absence of a comparator group and the occurrence of partial distal necrosis preclude definitive conclusions regarding superiority or comparative safety relative to fasciocutaneous or free-flap techniques. Larger prospective comparative studies with standardized functional and patient-reported outcome measures are warranted to better define the indications, limitations, and long-term outcomes of this technique.

## Figures and Tables

**Figure 1:**
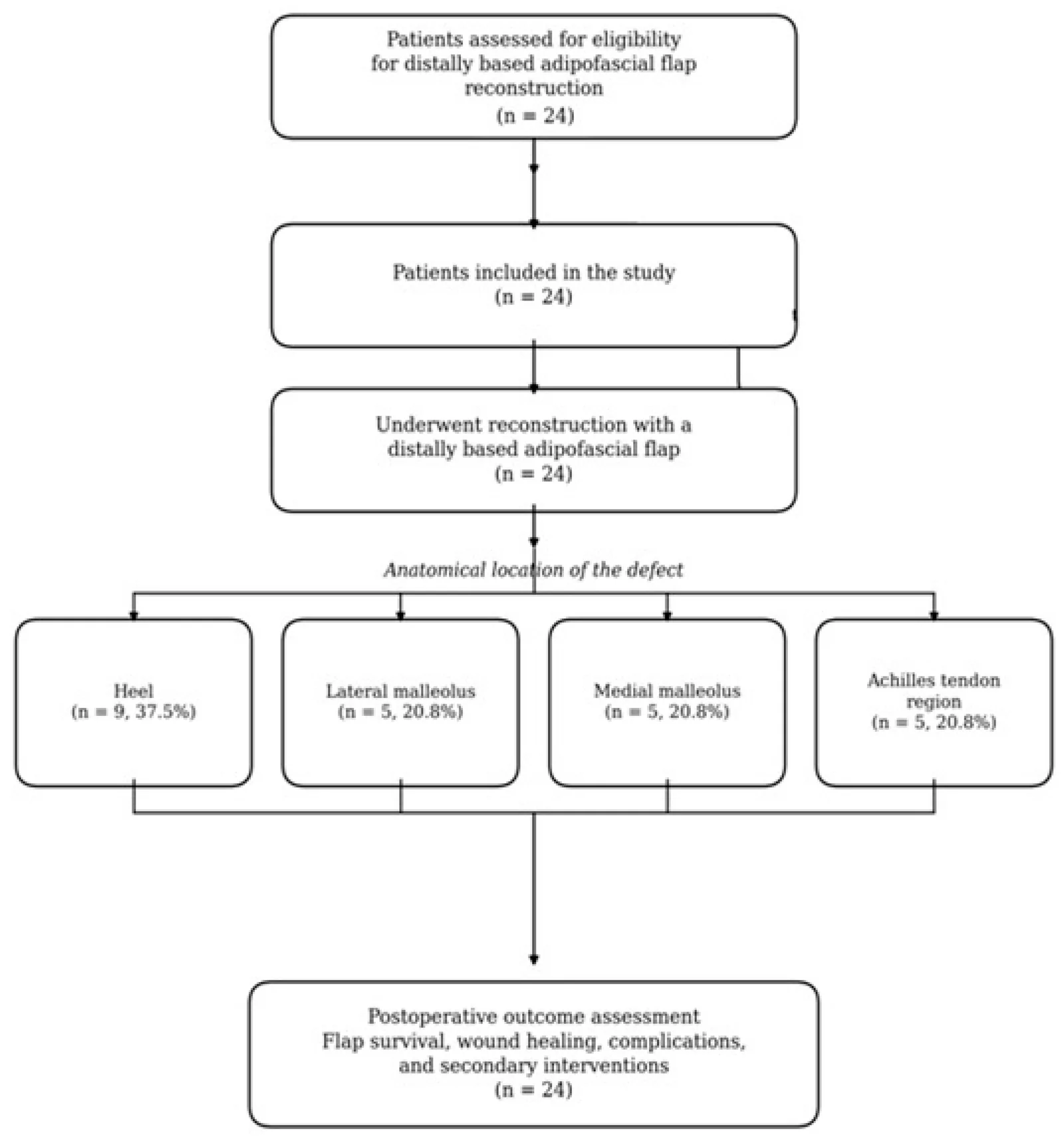
Study flow diagram summarizing patient selection and outcome assessment.

**Figure 2:**
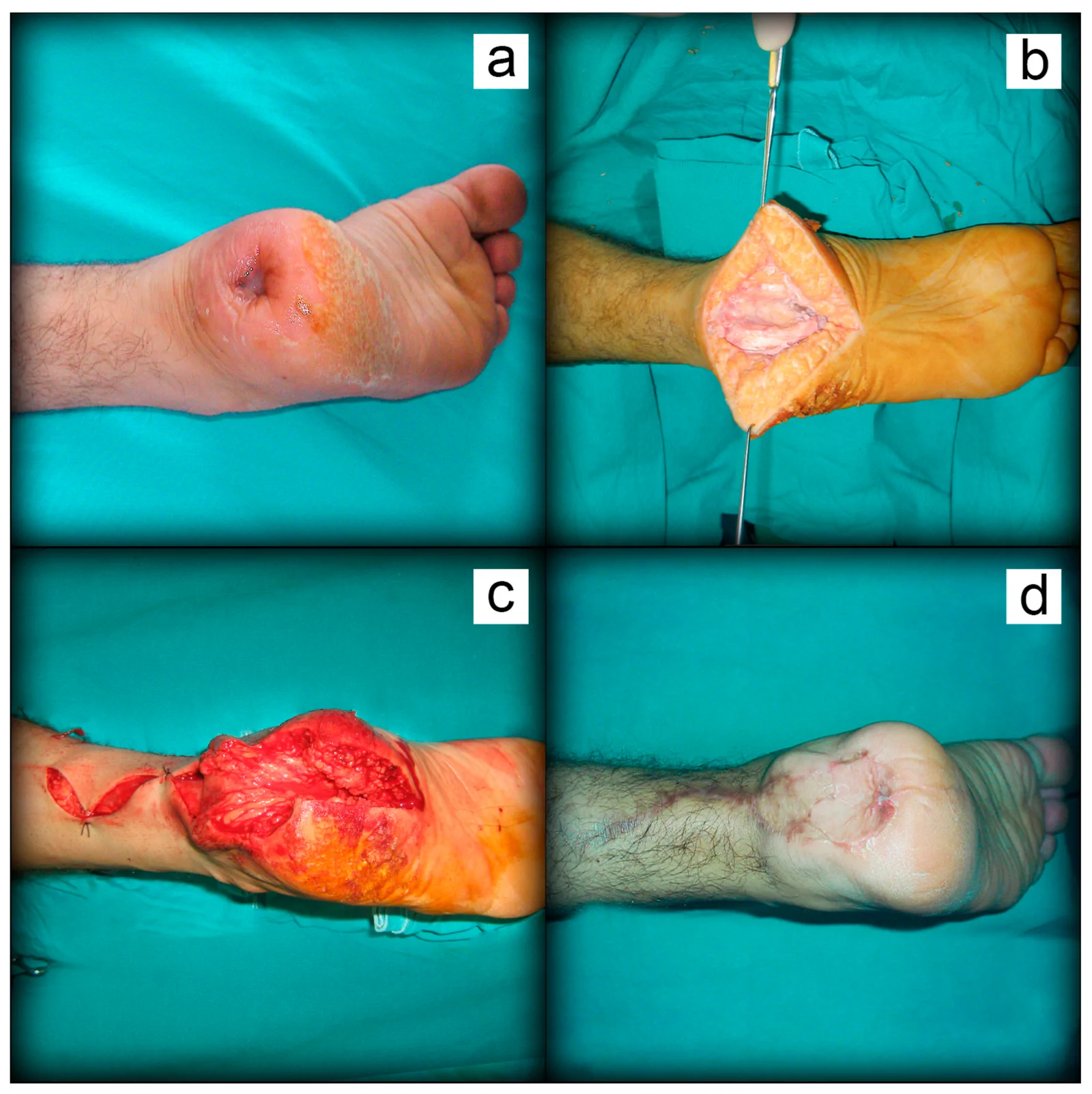
Reconstruction of a chronic plantar heel wound using a distally based adipofascial flap in a 54-year-old male patient. (**a**) Preoperative appearance of the chronic wound involving the weight-bearing plantar heel. (**b**) Intraoperative appearance of the defect following surgical debridement. (**c**) Elevation and distal transposition of the adipofascial flap to achieve tension-free coverage of the defect. (**d**) One-year postoperative appearance demonstrating complete wound healing, stable soft-tissue coverage, and satisfactory contour.

**Figure 3:**
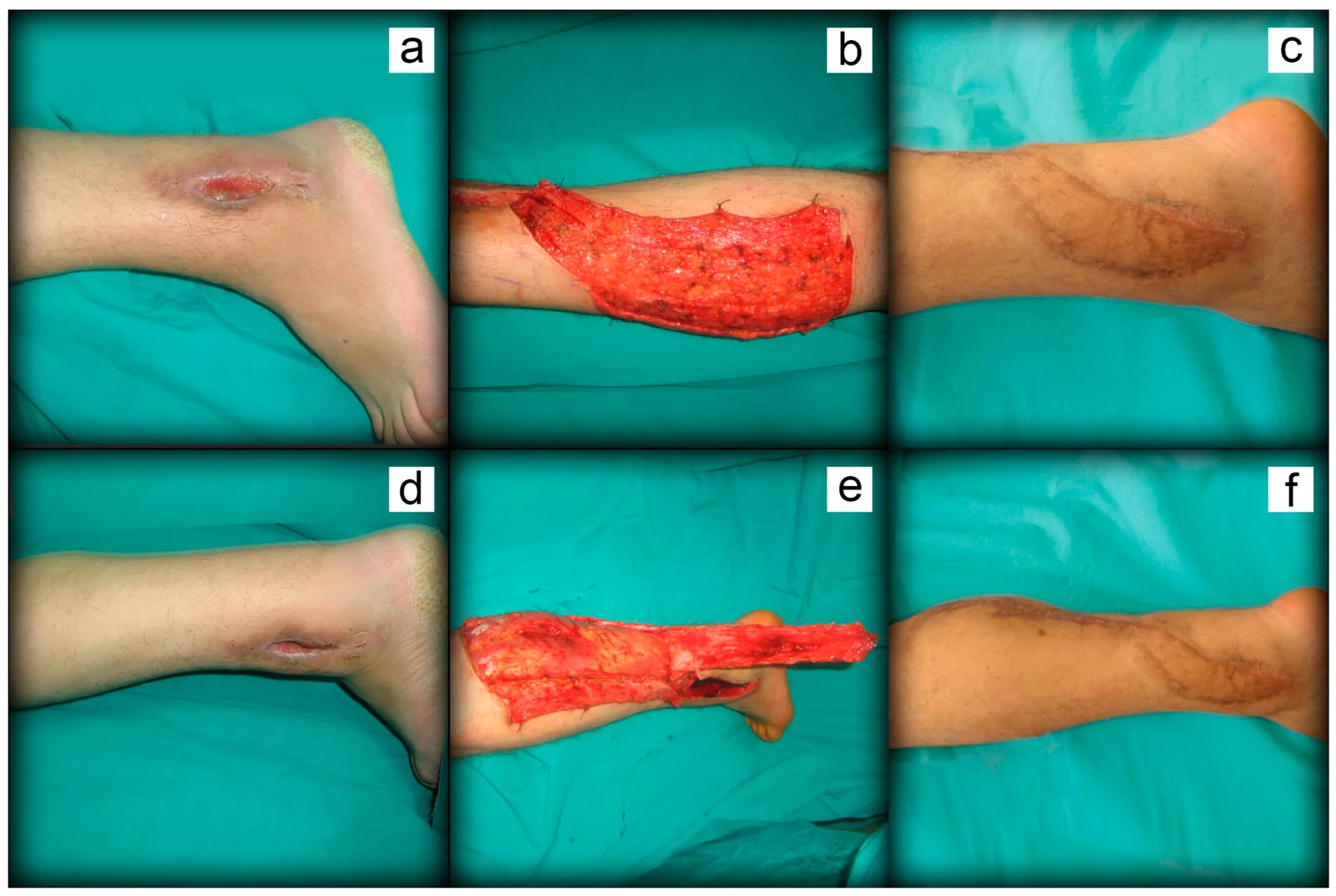
Reconstruction of a post-traumatic lateral malleolar defect using a distally based adipofascial flap in a 40-year-old male patient. (**a**,**d**) Preoperative views of the post-traumatic soft-tissue defect over the lateral malleolus. (**b**,**e**) Intraoperative views demonstrating elevation and distal transposition of the adipofascial flap toward the defect. (**c**,**f**) Postoperative views at 14 months demonstrating complete wound healing, stable soft-tissue coverage, and satisfactory contour.

**Figure 4:**
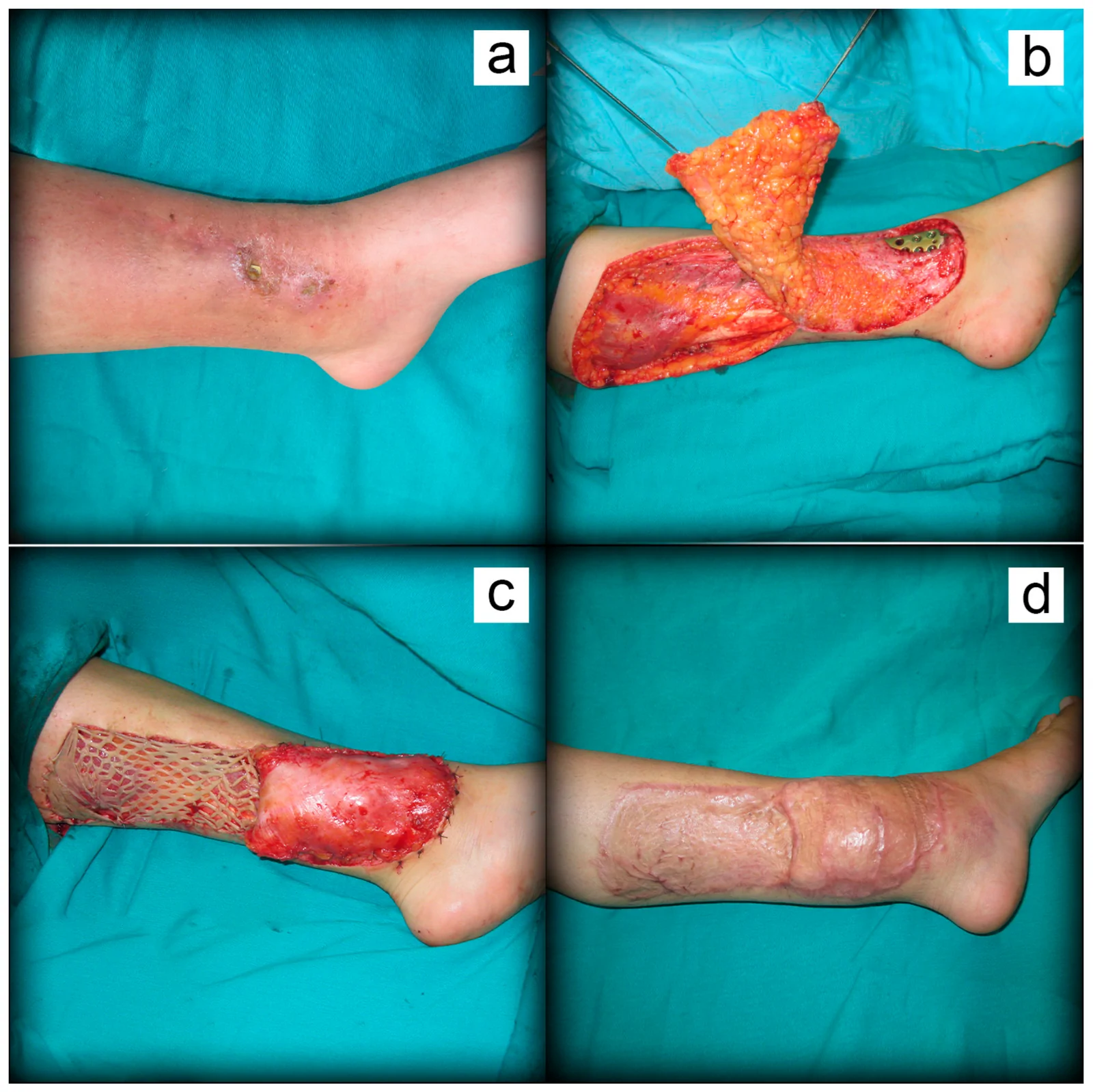
Reconstruction of a medial malleolar defect using a distally based adipofascial flap in a 42-year-old female patient. (**a**) Preoperative appearance of the medial malleolar wound with exposed orthopedic hardware. (**b**) Intraoperative view demonstrating elevation of the adipofascial flap and the defect overlying the fixation plate. (**c**) Flap inset and coverage with a split-thickness skin graft. (**d**) Postoperative appearance at 10 months demonstrating complete wound healing, stable coverage, and satisfactory contour.

**Figure 5:**
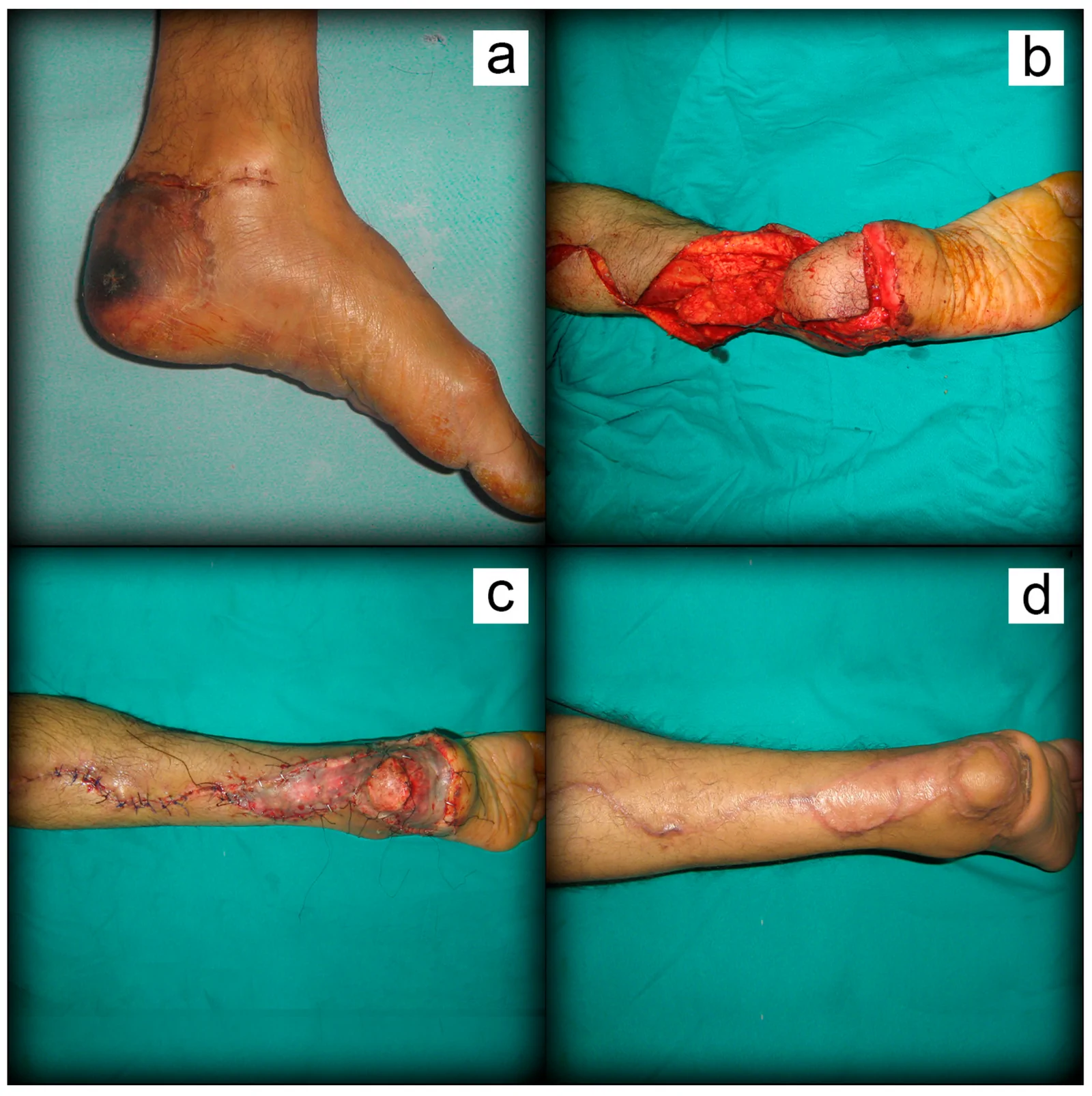
Reconstruction of a post-traumatic posterior heel defect using a distally based adipofascial flap in a 50-year-old male patient. (**a**) Preoperative appearance of the post-traumatic wound with soft-tissue necrosis over the posterior heel. (**b**) Intraoperative view demonstrating debridement of the nonviable tissue and elevation of the adipofascial flap. (**c**) Flap inset followed by split-thickness skin-graft coverage. (**d**) Postoperative appearance at 18 months demonstrating durable wound closure, stable soft-tissue coverage, and satisfactory contour.

**Figure 6:**
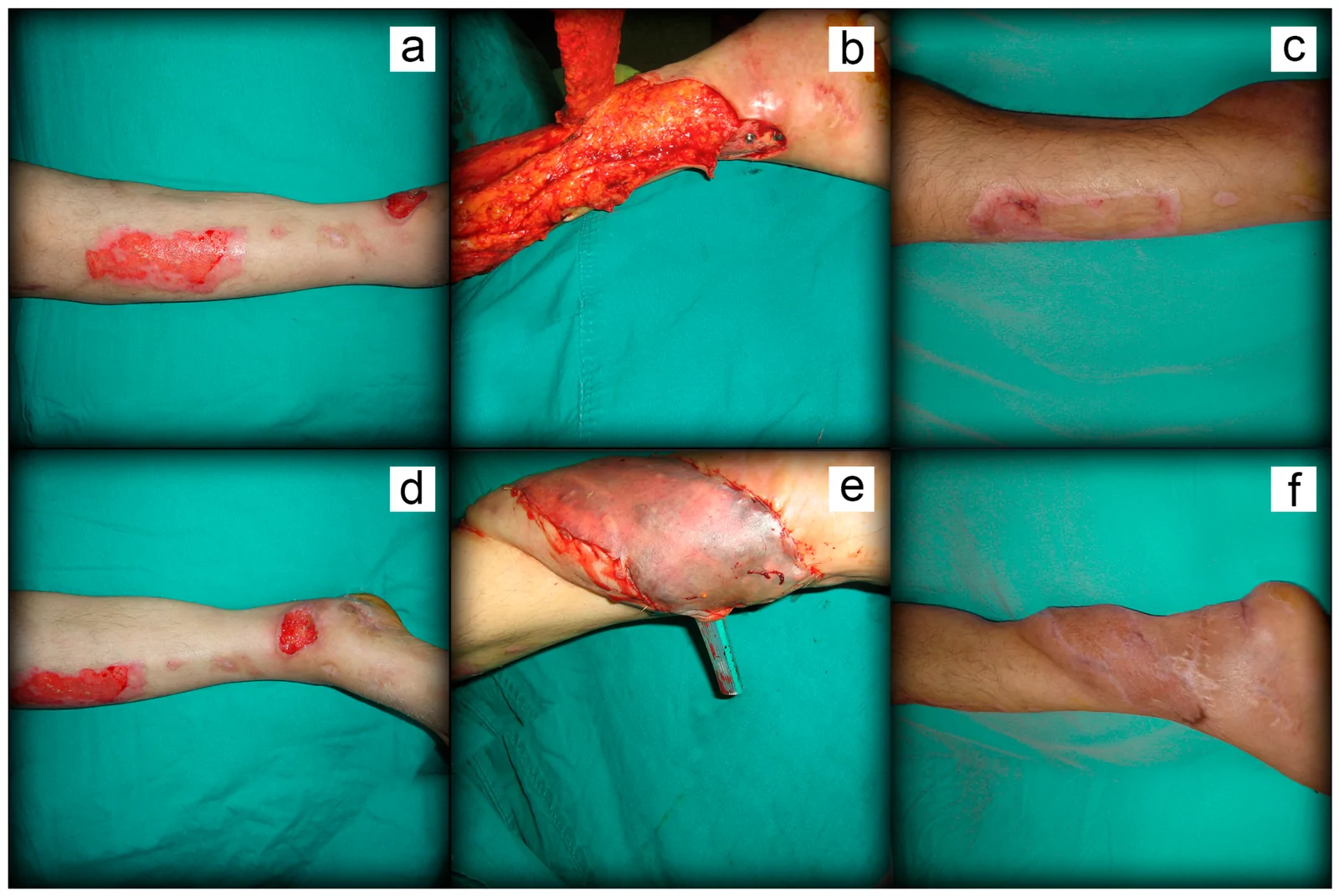
Reconstruction of post-traumatic distal lower-leg and ankle defects using a distally based adipofascial flap in a 30-year-old patient. (**a**,**d**) Preoperative views of the post-traumatic soft-tissue defects involving the distal lower leg and ankle. (**b**) Intraoperative view demonstrating flap elevation and the defect with exposed orthopedic hardware. (**e**) Flap inset and coverage with a split-thickness skin graft. (**c**,**f**) Postoperative views at 1 year demonstrating complete wound healing, stable soft-tissue coverage, and final contour.

**Table 1:** Demographic and Clinical Characteristics of the Study Population (*n* = 24).

Variable	Value
Sex, male/female	21 (87.5%)/3 (12.5%)
Age, years, range (mean)	30–64 (39)
Diabetes mellitus	6 (25.0%)
Underlying vascular disorder	4 (16.7%)
Defect etiology—chronic wound	11 (45.8%)
Defect etiology—trauma	8 (33.3%)
Defect etiology—infection	3 (12.5%)
Defect etiology—tumor excision	2 (8.3%)
Defect dimensions, cm, range	2.1–5.8
Duration of hospitalization, days, range	2–7
Postoperative immobilization, days, range	14–21

**Table 2:** Anatomical Distribution of Distal Lower-Extremity Defects (*n* = 24).

Anatomical Location	*n*	%
Heel	9	37.5
Lateral malleolus	5	20.8
Medial malleolus	5	20.8
Achilles tendon region	5	20.8
Total	24	100

**Table 3:** Postoperative Outcomes and Complications (*n* = 24). STSG, split-thickness skin graft.

Outcome/Complication	*n*	%	Management
Total flap loss	0	0	—
Distal flap-edge necrosis	5	20.8	Debridement + STSG (*n* = 3); secondary intention (*n* = 2)
Wound dehiscence	3	12.5	Secondary suturing
Postoperative local infection	2	8.3	Local wound care + oral antibiotics

## Data Availability

The data presented in this study are available on request from the corresponding author due to patient privacy restrictions.

## Abbreviations

The following abbreviations are used in this manuscript:

STROBE	Strengthening the Reporting of Observational Studies in Epidemiology
STSG	Split-thickness skin graft
